# Supplementary material for: Large haploblocks underlie rapid adaptation in the invasive weed Ambrosia artemisiifolia
Source: Nat Commun. 2023 Mar 27;14:1717. doi: 10.1038/s41467-023-37303-4 (PMC10042993; doi:10.1038/s41467-023-37303-4)
Supplement: Supplementary file 1 — Supplementary Information [file 41467_2023_37303_MOESM1_ESM.pdf]

Large haploblocks underlie rapid adaptation in the invasive weed *Ambrosia artemisiifolia*

Battlay *et al.*

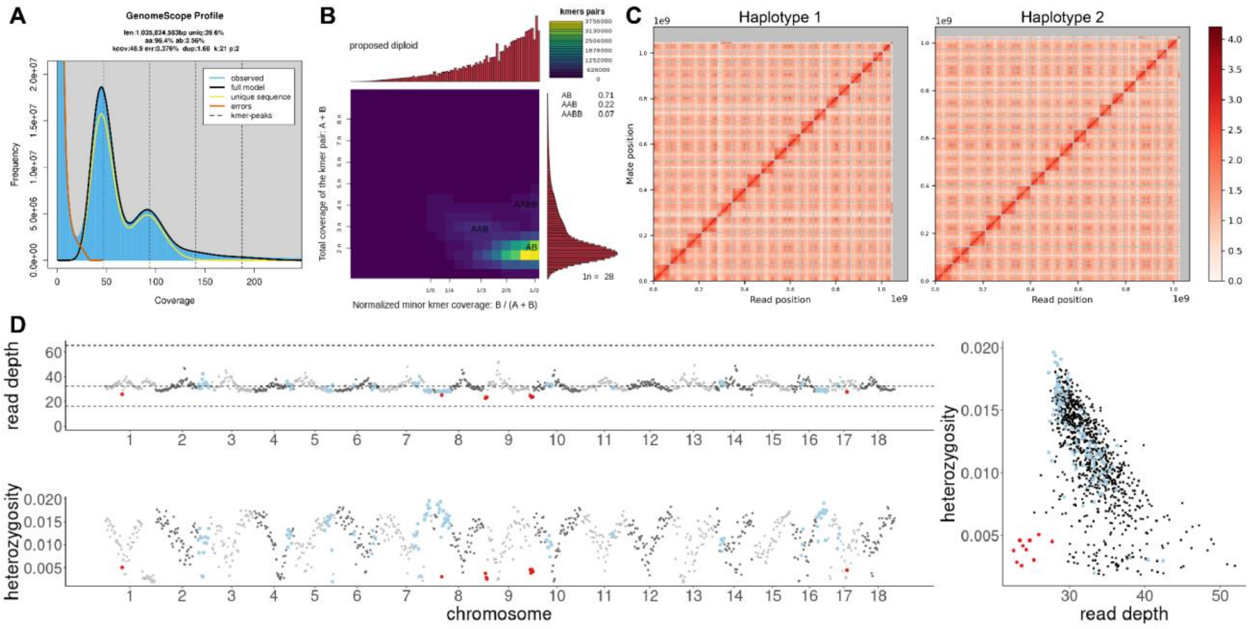

Supplementary Figure 1. ***Ambrosia artemisiifolia* genome assembly quality. A.** The kmer coverage and model fit from GenomeScope 2.0 for 21mers from the PacBio HiFi and OmniC Illumina reads. **B.** A smudgeplot used to estimate ploidy from heterozygous kmer pairs using 21mers from the HiFi reads. **C.** Link density histograms, identified by proximity ligation sequencing for haplotype 1 and haplotype 2. The *x* and *y* axes show mapping positions of the first and second read in read pairs. **D.** Read depth and heterozygosity in 1Mbp windows for Illumina reads used in the reference genome assembly mapped to the final version of the reference genome. Windows in the bottom 10% of read depth and heterozygosity values are indicated in red; windows overlapping haploblocks are indicated in pale blue.

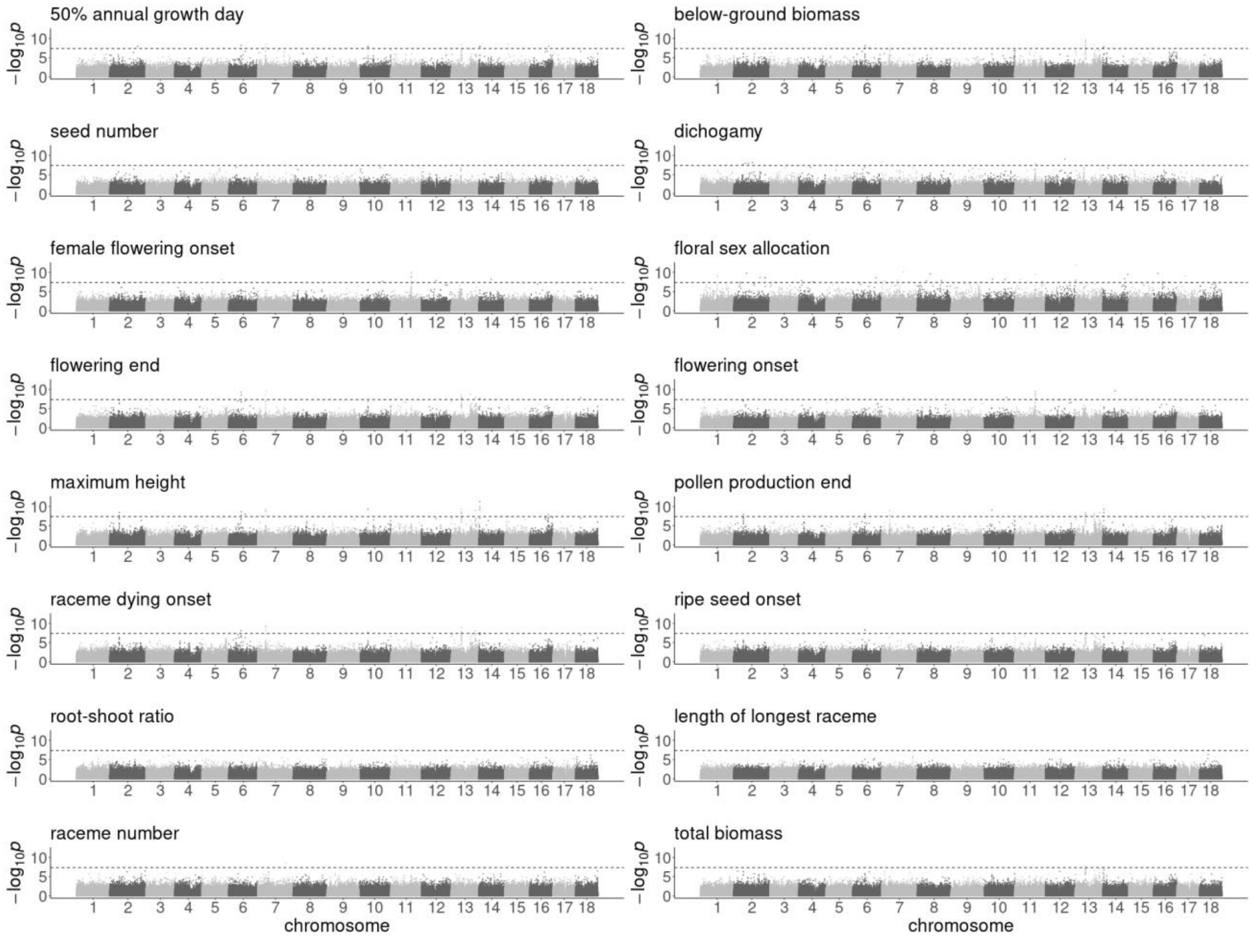

Supplementary Figure 2. Genome-wide association study results for phenotypes with significant SNPs. -$\log_{10}p$-value of mixed model association against genomic location. Solid lines indicate a bonferroni-corrected significance threshold of 0.05

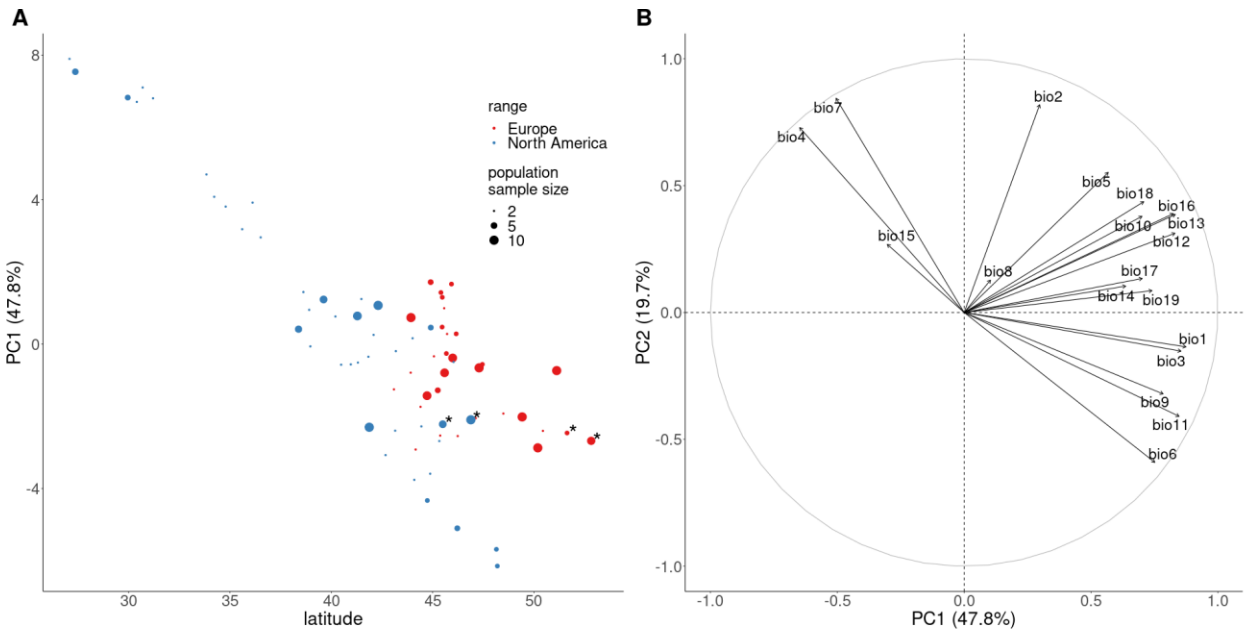

Supplementary Figure 3. Environmental comparison of North American and European *Ambrosia artemisiifolia* ranges. A. The first principle component of 19 bioclimatic variables against latitude for modern ragweed populations in North America (red) and Europe (blue), with asterisks identifying populations with high *ELF3* allele frequencies. **B**. A variable correlation plot for 19 bioclimatic variables.

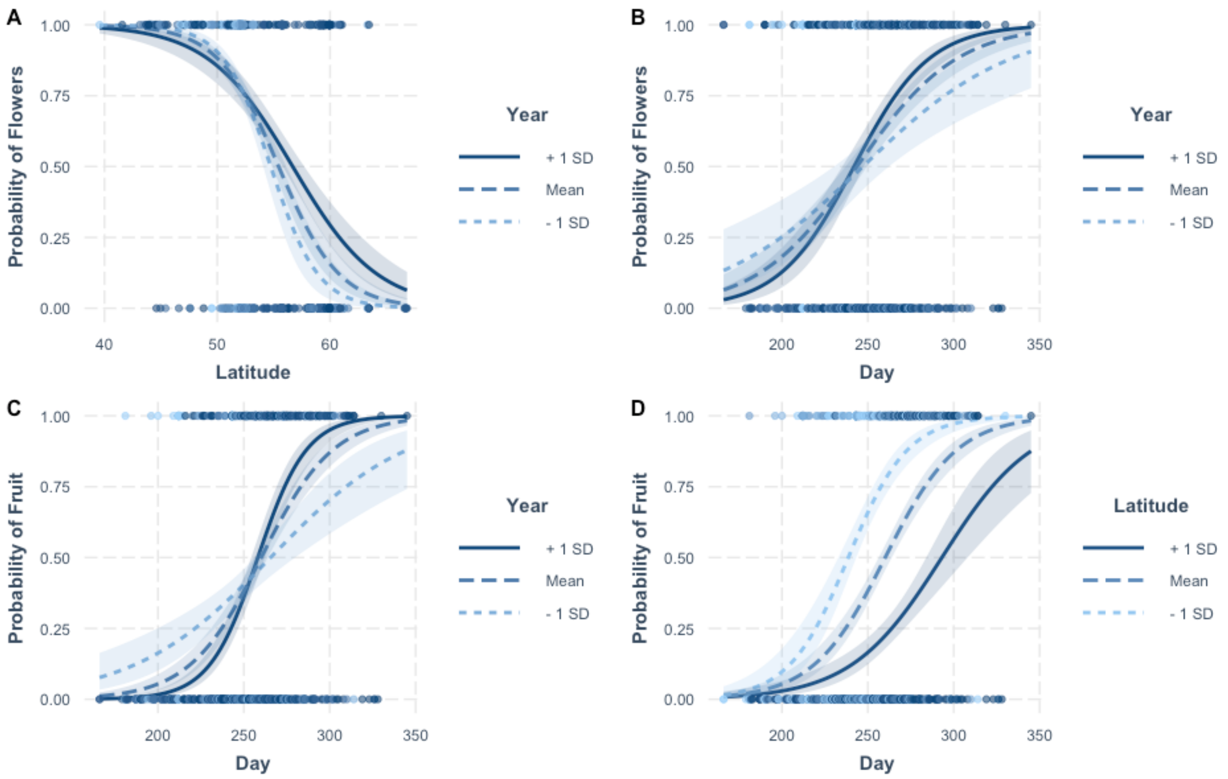

Supplementary Figure 4. Phenology shifts over time in European herbarium specimens. Interaction plots illustrating the results of generalized linear models examining the presence of mature male inflorescences (probability of flowers) or mature fruit (probability of fruit) in herbarium specimens of *A. artemisiifolia* in Europe as a function of collection day (Day), latitude of origin (Latitude) and collection year (Year). The predicted probability of observing flowers is plotted as a function of latitude (**A**), or collection day (**B**) for different collection years (mean collection year +/- 1 SD). The predicted probability of observing fruit is plotted against collection day for different collection years (mean collection year +/- 1 SD; **C**) or latitudes (mean collection latitude +/- 1 SD; **D**). Confidence intervals for the predictions are shown as are the raw data.

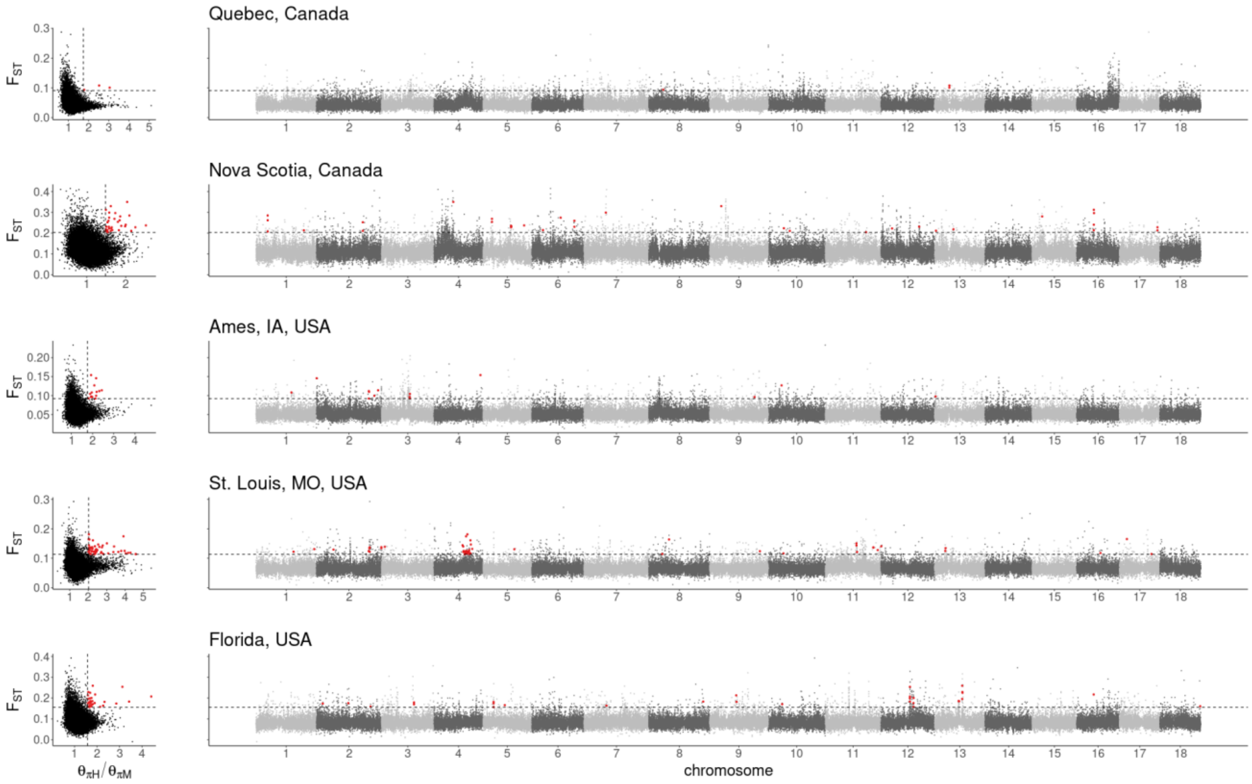

Supplementary Figure 5. Distributions of F_{ST} and $\theta_{\pi H}/\theta_{\pi M}$ between historic and modern samples from North American populations, and F_{ST} against genomic location. Red points indicate putative selective sweep windows, which are in top one percent of per-window F_{ST} and $\theta_{\pi H}/\theta_{\pi M}$ (dashed lines).

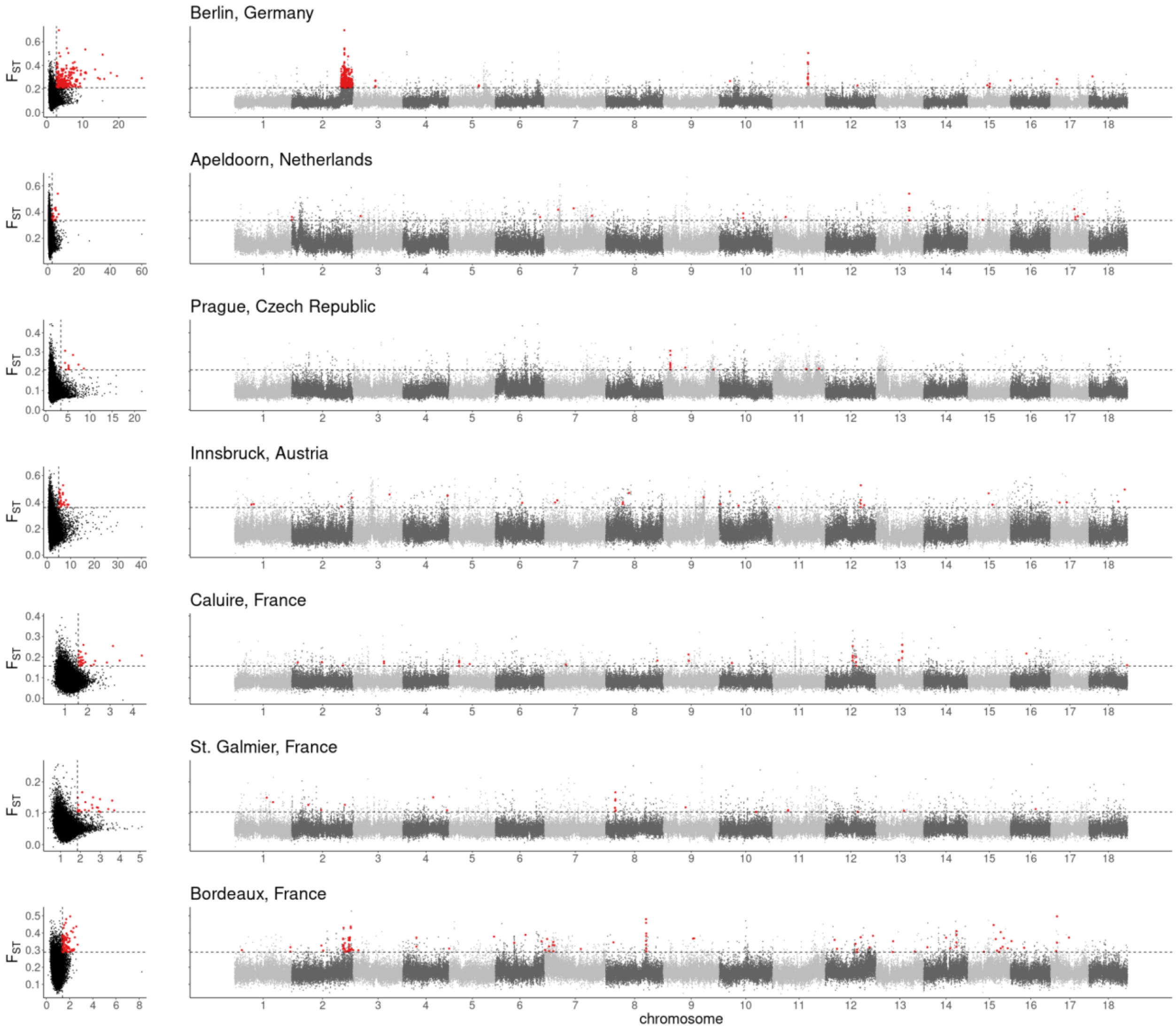

Supplementary Figure 6. Distributions of F_{ST} and $\theta_{\pi H}/\theta_{\pi M}$ between historic and modern samples from European populations, and F_{ST} against genomic location. Red points indicate putative selective sweep windows, which are in top one percent of per-window F_{ST} and $\theta_{\pi H}/\theta_{\pi M}$ (dashed lines).

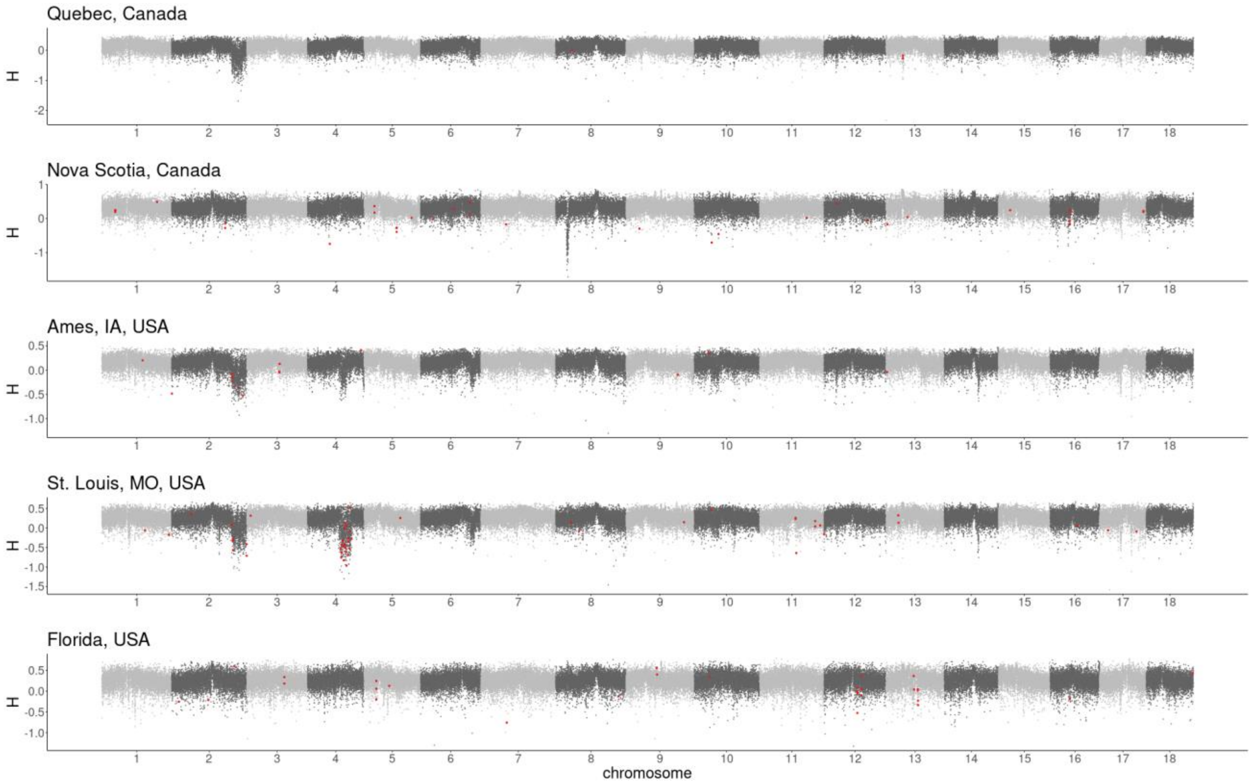

Supplementary Figure 7. Fay and Wu's *H* against genomic location for modern North American populations. Red points indicate putative selective sweep windows from historic-modern comparisons.

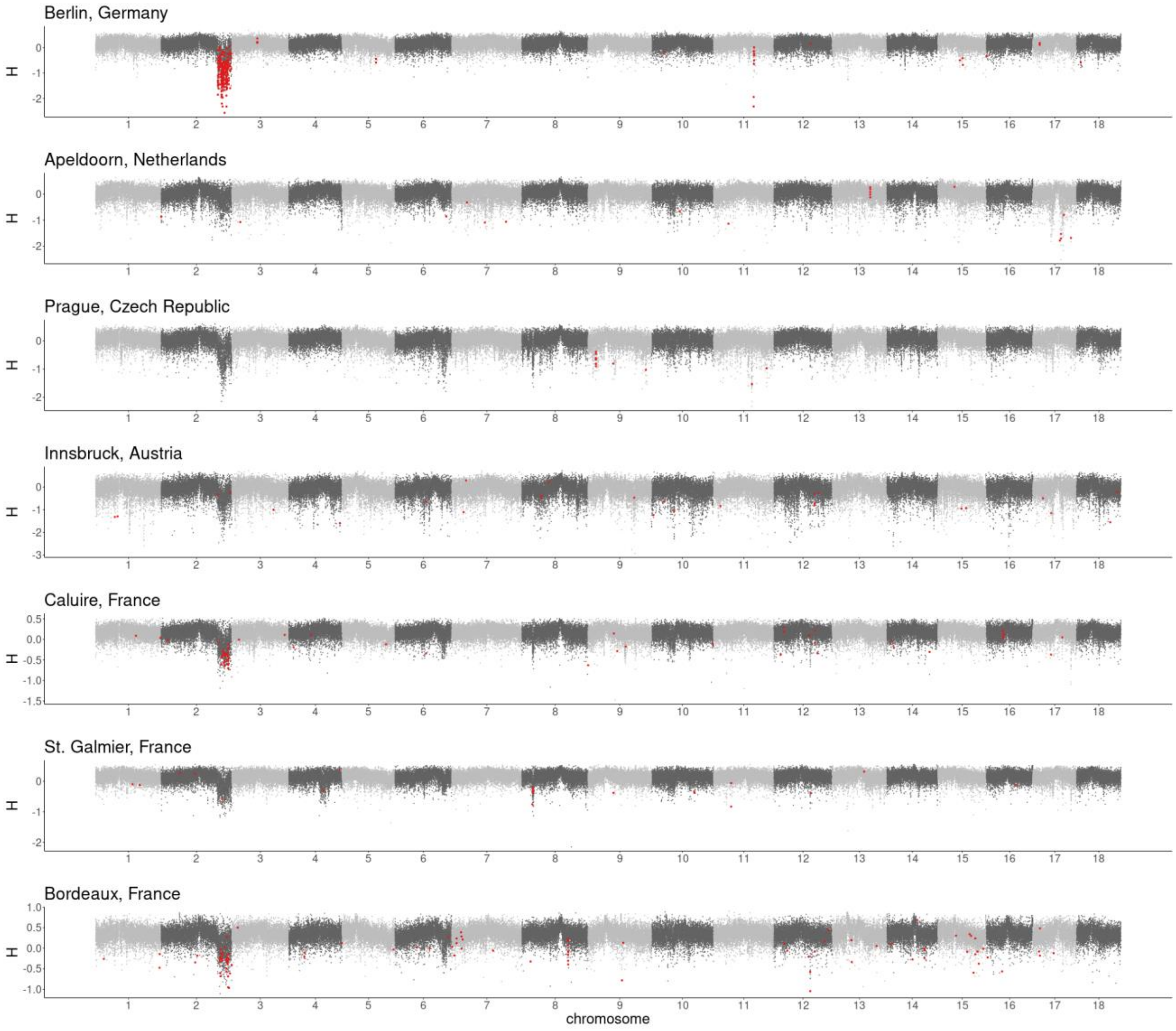

Supplementary Figure 8. Fay and Wu's *H* against genomic location for modern European populations. Red points indicate putative selective sweep windows from historic-modern comparisons.

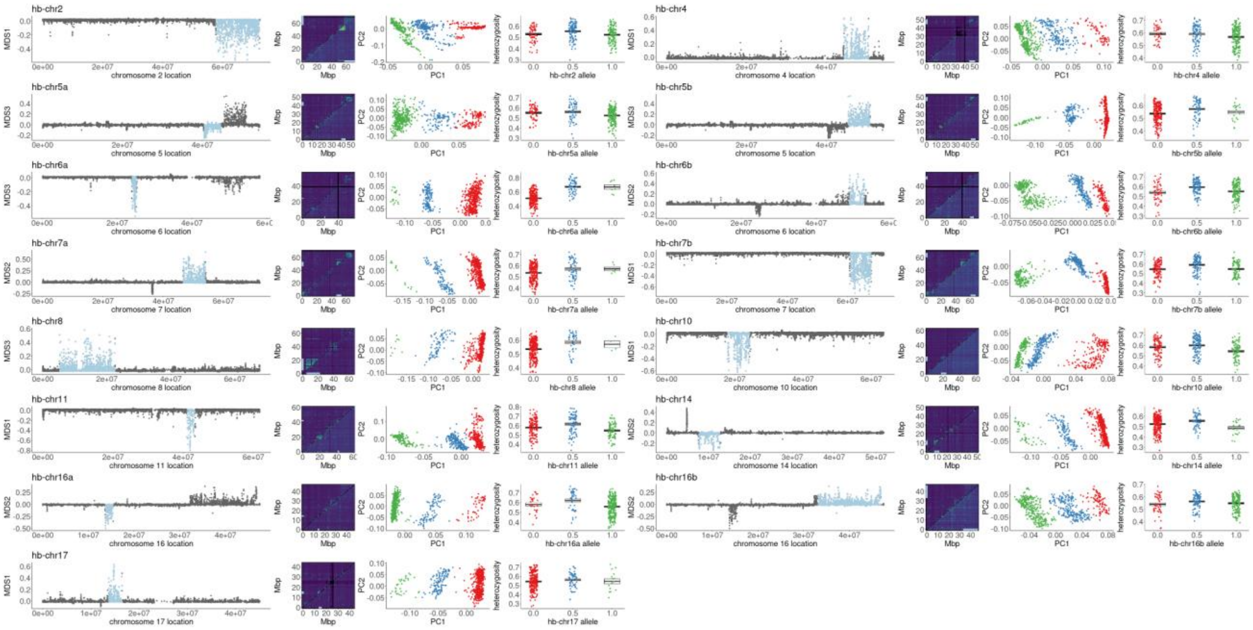

Supplementary Figure 9. Evidence that chromosomal inversions underlie haploblock signatures. Haploblocks display extreme, divergent, local population structure (pale blue regions; first column). Haploblock regions (indicated by pale blue lines; second column) correspond to blocks of linkage disequilibrium (second highest r^2 in 0.5Mb windows) apparent using all modern samples (top triangle) but often reduced or absent using only samples homozygous for the more common haploblock genotype (bottom triangle). Haploblock genotypes were assigned by kmeans clustering (colours; third column) using the first two principal components of genetic variation across haploblock regions. Heterozygous haploblock genotypes show elevated mean per-site heterozygosity (fourth column; *n*=311 biologically independent samples; boxes denote mean +/- SEM in each cluster).

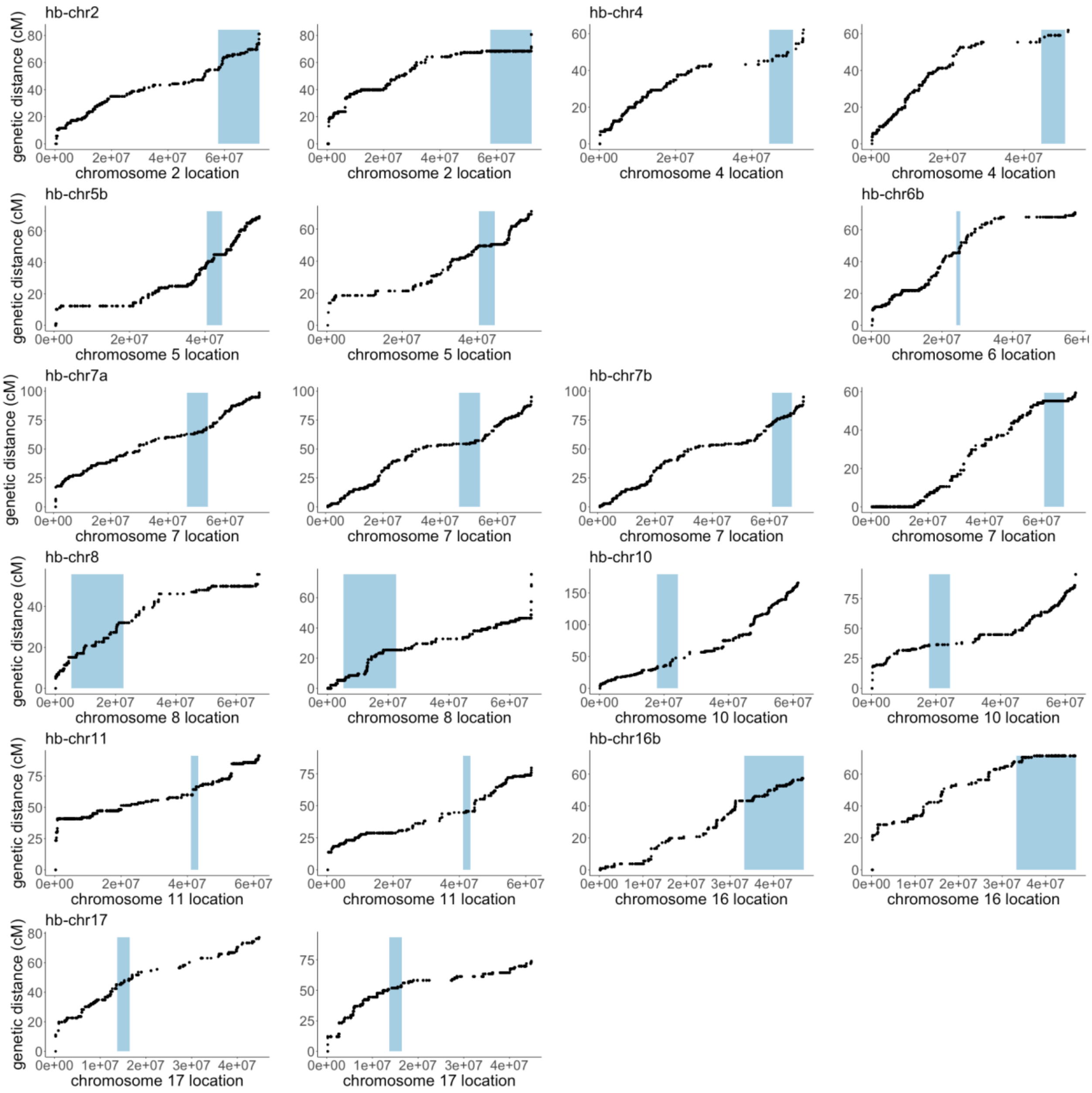

Supplementary Figure 10. Evidence of genotype-specific reductions in recombination in haploblock regions. Genetic distance (cM) against physical distance (bp) along a portion of each scaffold is shown. Haploblock regions are shown in pale blue. Example maps are displayed showing both low recombination rates (left) and high recombination rates (right) for each haploblock.

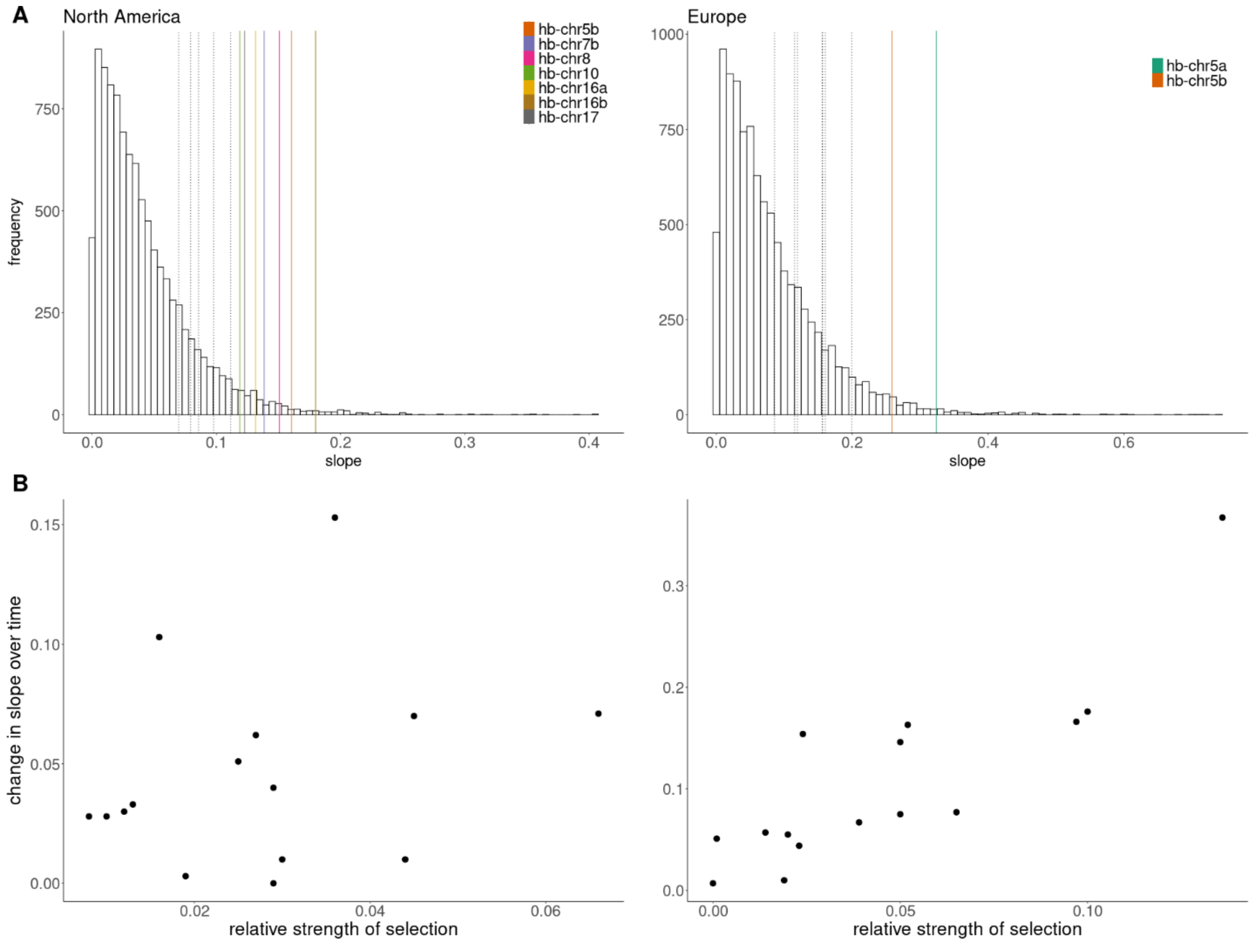

Supplementary Figure 11. Analysis of haploblock slope estimates. A. The distribution of slope estimates from generalized linear models of population allele counts against latitude for 10,000 randomly selected SNPs in each range. The vertical lines show the slope estimates for haploblocks with statistical associations with latitude in one range (Supplementary Table 8). Solid lines represent estimates in the 5% tail of each distribution while dotted lines fall below that cut-off. **B.** The change in the slope of the relationship between latitude and haplotype frequency (see Supplementary Data 6) between historic and modern samples compared to the estimate of selection along the latitudinal cline for the 15 haploblocks (estimated from modern data in each range). A strong relationship was detected in the invasive European (r_{13} = 0.86, t = 5.99, $p = 4.55\times10^{-5}$, 95% CI = {0.61, 0.95}), but not the native North American range (r_{13} = 0.25, t = 0.95, p = 0.36, 95% CI = {-0.30, 0.68}).

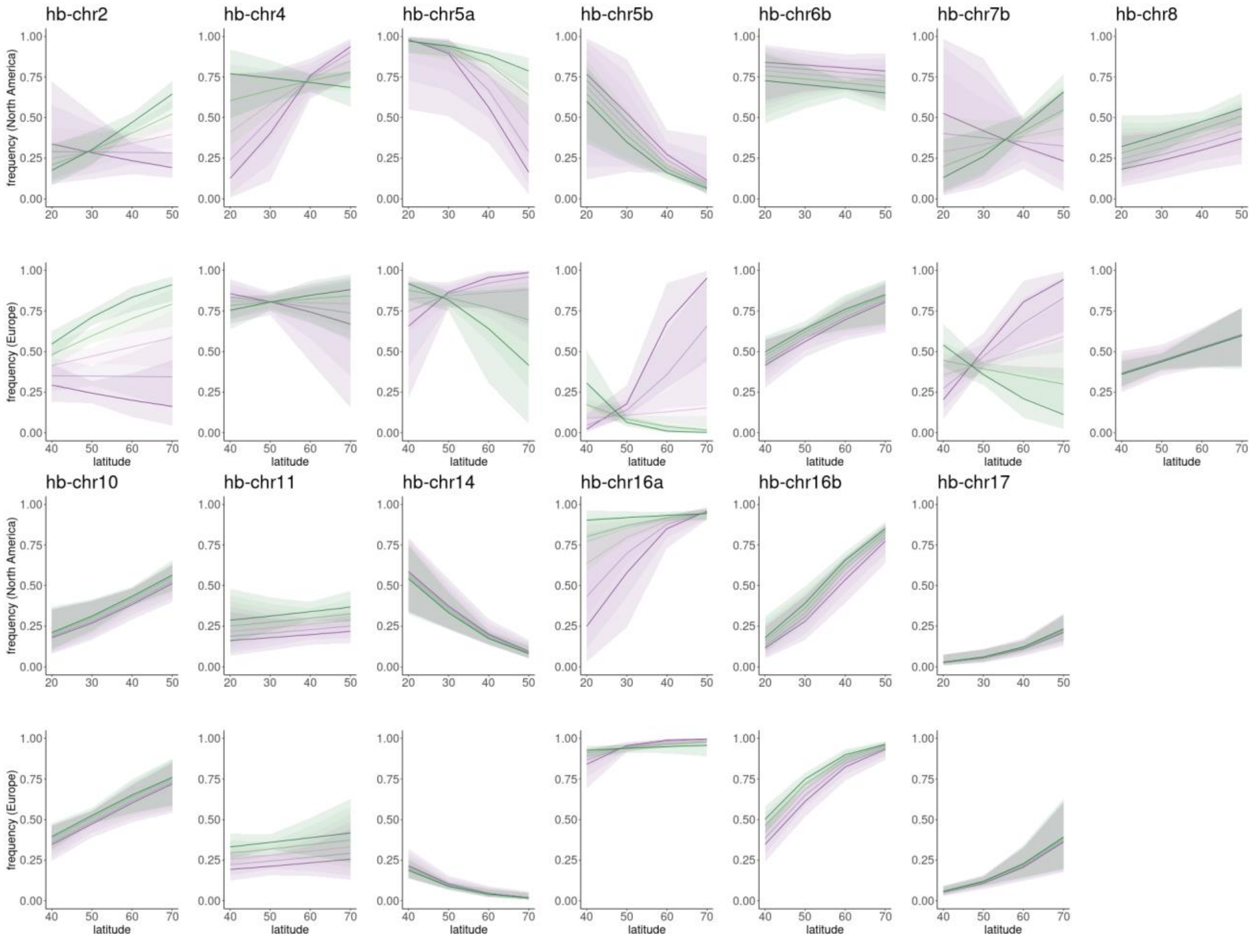

Supplementary Figure 12. Haploblock frequency changes over space and time. Logistic regression models with 95% CI ribbons (see Supplementary Table 9 and Supplementary Data 5-9 for model details) of haploblock frequency (allele 1) against latitude for each haploblock that shows a significant latitude, time or range effect, or significant interactions between these effects, across five time bins ranging from most historic (purple) to most modern (green).

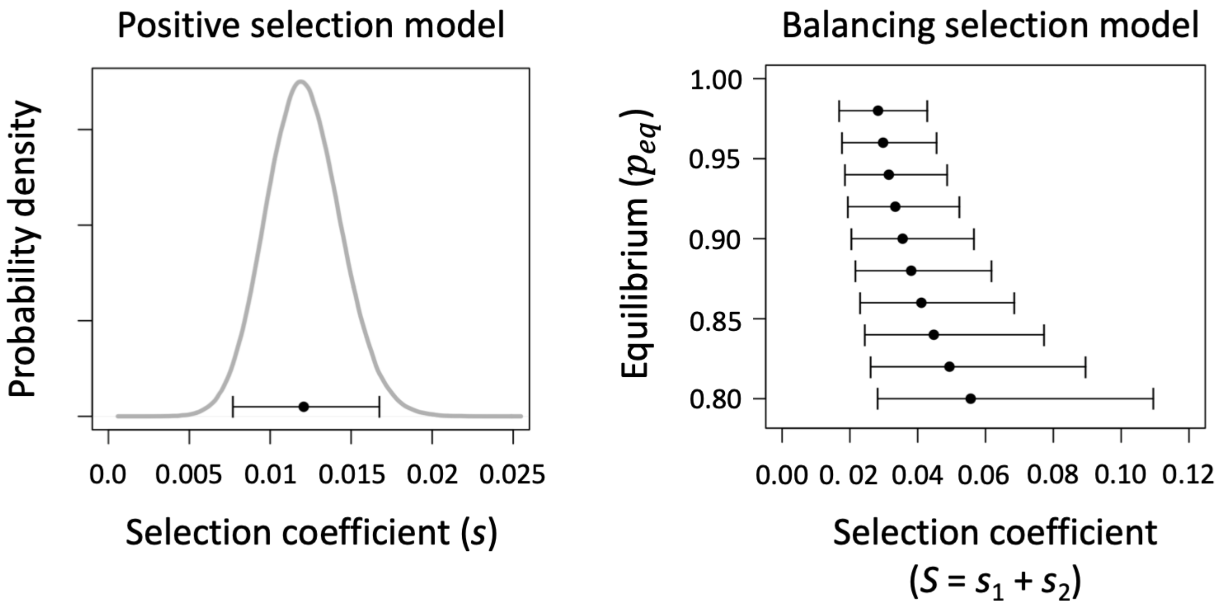

Supplementary Figure 13. Selection coefficients consistent with observed frequency changes of the hb-chr2 haplotype under models of positive selection and balancing selection. The left-hand panel shows the distribution of *s* values (gray) and the 95% CI for *s* (in black: parallel to the *x*-axis), consistent with temporal change in the hb-chr2 haploblock. The distribution of *s* is based on 10^6 simulations of initial and final frequencies of the haploblock that are consistent with the estimated frequencies and error in the estimates. For each simulated value of *s*, we first simulated initial and final haplotype frequencies as described in Supplementary Methods 1. Supplementary Eq. (**2**) was used to calculate the value of *s* consistent with each combination of initial and final frequencies, and using a time interval between 1902 (the median year of historic samples used in this analysis) and 2014 (contemporary). The right-hand panel shows the selection coefficients consistent with observed changes in hb-chr2 inversion frequencies in Europe, assuming a model of balancing selection as described in Supplementary Methods 1. Haplotype frequencies were simulated as in the positive selection model and selection coefficients ($S = s_1 + s_2$) were calculated from Supplementary Eq. (**5**). Across the range of possible polymorphic equilibrium frequencies under balancing selection, equilibria near unity (*e.g.*, p_{eq} = 0.98) require modestly strong selection (average S = 0.028; 95% CI = [0.017, 0.043]) to explain the observed frequency changes in hb-chr2, and lower equilibrium states require stronger selection.

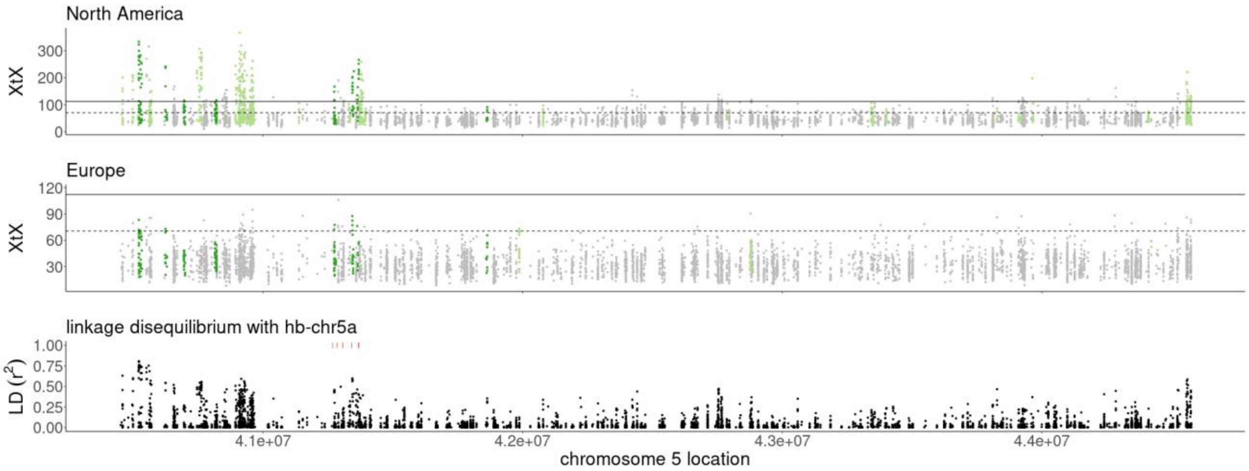

Supplementary Figure 14. A detailed analysis of the hb-chr5 region (5:40446189-44576095). XtX and XtX-EAA outlier windows zoomed from Fig. 2C, and linkage disequilibrium (r^2) between SNPs in the region and hb-chr5a haploblock genotype. A cluster of six pectate lyase genes, consisting of the top BLAST hit for *Amba1* and closely-related paralogues, are indicated in red above the linkage disequilibrium plot.

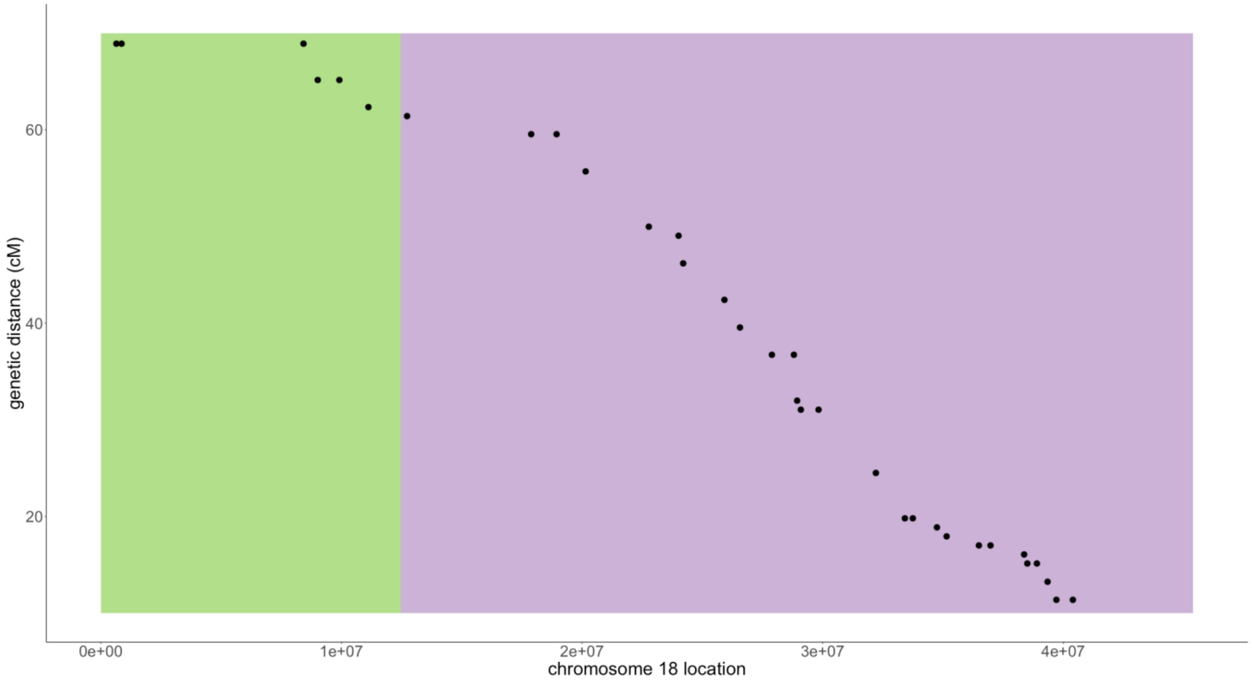

Supplementary Figure 15. Assembly of haplotype 1 chromosome 18. Genetic markers support the combination of two scaffolds (green and purple).

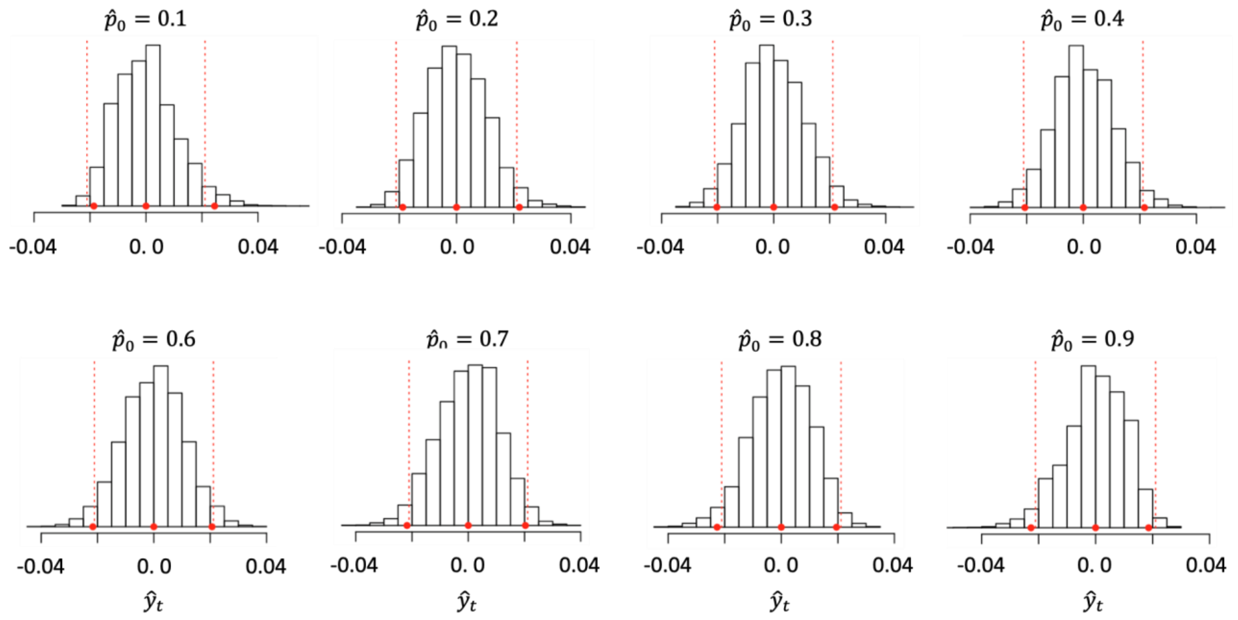

Supplementary Figure 16. Simulated distributions of scaled divergence given different initial allele frequencies. Each distribution is based on 10^4 independent and neutrally evolving SNPs. The simulations use n_0 = 182, n_t = 312, and t = 131, with a moderate effective population size (N_e = 10^4). Histograms show the distribution of $\hat{y}_t$ estimates, the red circles show the simulated mean and 95% confidence intervals for simulated data, and the vertical broken red lines show +/- 1.96 SD, where SD is the square root of our analytical expression for $var[\hat{y}_t]$.

Supplementary Table 1. ***Ambrosia artemisiifolia*** **phased diploid genome assembly statistics.**

Statistics	Haplotype 1	Haplotype 1 (chromosomes)	Haplotype 2	Haplotype 2 (chromosomes)
L50	9	8	9	8
N50 (Mbp)	58.5	59.6	58.04	60.5
L75	14	13	14	13
N75 (Mbp)	52.3	54.4	49.7	52.5
Largest contig (Mbp)	72.4	72.4	72.3	72.3
n contigs	1498	18	747	18
Genome size (Gbp)	1.11	1.05	1.07	1.03
Assembled in chromosomes (%)	94.3	100	96.5	100
BUSCO* (*n*)	178:77:0:0	183:72:0:0	181:74:0:0	183:72:0:0
BUSCO* (%)	69.8:30.2:0:0	71.8:28.2:0:0	71:29:0:0	71.8:28.2:0:0

*Number of BUSCO (Benchmarking Universal Single-Copy Ortholog) genes found in the assembly using the *eukaryota odb10* dataset. Genes are split into four categories: complete and single-copy, complete and duplicated, fragmented, and missing, and scores are reported in this order.

Supplementary Table 2. ***Ambrosia artemisiifolia*** **phased diploid genome assembly chromosome sizes.**

Chromosome	Haplotype 1 length (bp)	Haplotype 2 length (bp)
1	67,187,999	66,124,883
2	72,449,833	72,311,999
3	58,521,165	61,691,755
4	54,534,567	44,211,056
5	54,440,431	53,910,277
6	58,073,023	58,035,721
7	72,096,067	71,854,087
8	67,786,512	66,872,696
9	65,108,644	55,085,163
10	63,683,632	61,943,115
11	62,034,392	60,512,409
12	59,565,153	60,716,626
13	56,271,462	55,506,995
14	52,313,486	52,489,210
15	49,799,525	49,691,924
16	47,551,965	49,669,858
17	45,018,532	43,698,553
18	45,402,704	44,461,152

Supplementary Table 3. Summary of annotations of protein coding genes.

Primary transcripts	36,826
Mean exon number per gene	5.75
Median transcript length (bp)	3009
Median CDS length (bp)	969
Median exon length (bp)	235
Median intron length (bp)	399
Median 3' UTR length (bp)	229
Median 5' UTR length (bp)	130

Supplementary Table 4. Environmental correlation outlier windows that are also XtX outlier windows for each range, and for parallel windows between ranges.

Test	North America	Europe	Parallel
XtX	2704	2697	640
All variables	2167	1357	291
Annual mean temperature	684	158	14
Mean diurnal range	237	152	3
Isothermality	408	168	12
Temperature seasonality	968	63	5
Max. temperature of warmest month	490	59	2
Min. temperature of coldest month	835	246	21
Temperature annual range	1065	83	5
Mean temperature of wettest quarter	570	188	16
Mean temperature of driest quarter	843	268	24
Mean temperature of warmest quarter	588	60	2
Mean temperature of coldest quarter	781	276	26
Annual precipitation	1105	291	43
Precipitation of wettest month	951	260	34
Precipitation of driest month	1060	283	52
Precipitation seasonality	1004	254	30
Precipitation of wettest quarter	904	269	35
Precipitation of driest quarter	1045	302	52
Precipitation of warmest quarter	918	273	29
Precipitation of coldest quarter	1058	292	48

Supplementary Table 5. Two-sided Fisher's exact test results for enrichment of flowering time pathway genes in XtX-EAA outlier windows, and in haploblocks.

Outlier windows	*p*-value
North America XtX-EAA	0.0074 **
Europe XtX-EAA	0.06
Parallel XtX-EAA	0.49
All haploblocks	0.0018 **
hb-chr2	0.065
hb-chr4	0.22
hb-chr5a	0.09
hb-chr5b	0.0010 **
hb-chr6a	1
hb-chr6b	1
hb-chr7a	1
hb-chr7b	0.83
hb-chr8	0.14
hb-chr10	0.3
hb-chr11	0.15
hb-chr14	0.36
hb-chr16a	1
hb-chr16b	0.73
hb-chr17	0.6

Supplementary Table 6. The results of the generalized linear model examining the presence of mature male inflorescences (binary trait) in herbarium specimens *Ambrosia artemisiifolia* in Europe. Day of collection, latitude of origin, and collection year as well as all interactions were included in the initial model and non-significant interactions were removed sequentially, starting with the highest order interaction. McFadden's pseudo-R-squared for the reduced model was 0.26.

Effect	Degrees of freedom	*F*-value	*p*-value
Day	1, 886	7.83	0.005129 **
Latitude	1, 886	8.85	0.003005 **
Year	1, 886	21.19	4.768e-06 ***
Day:year	1, 886	8.83	0.003037 **
Latitude:year	1, 886	7.89	0.005083 **

Supplementary Table 7. The results of the generalized linear model examining the presence of fruit (binary trait) in herbarium specimens *Ambrosia artemisiifolia* in Europe. Day of collection, latitude of origin, and collection year as well as all interactions were included in the initial model and non-significant interactions were removed sequentially, starting with the highest order interaction. McFadden's pseudo-*R*-squared for the reduced model was 0.24.

Effect	Degrees of freedom	*F*-value	*p*-value
Day	1, 886	28.58	1.146e-07 ***
Latitude	1, 886	1.19	0.27607
Year	1, 886	33.68	9.054e-09 ***
Day:latitude	1, 886	4.28	0.03885 *
Day:year	1, 886	32.33	1.763e-08 ***

Supplementary Table 8. Temporal signatures of selective sweeps in individual populations, and their overlap with spatial signatures of selection.

Population	Historic *n*	Modern *n*	Historic year	Modern year	Sweep windows	Range XtX-EAA	Parallel XtX-EAA
**North America**					**129**	**9**	**3**
Ames, IA, USA	5	10	1896-1904	2009	14	2	1
Florida, USA	5	5	1913-1930	2009	29	0	0
Nova Scotia, Canada	4	4	1921	2009	33	2	1
Quebec, Canada	10	10	1928-1938	2013	3	0	0
St. Louis, MO, USA	4	6	1875-1891	2009	50	5	1
**Europe**					**476**	**17**	**5**
Apeldoorn, Netherlands	10	10	1875-1895	2014	21	0	0
Berlin, Germany	7	8	1872-1875	2017	251	13	5
Bordeaux, France	8	4	1920-1930	2019	98	1	0
Caluire, France	18	10	1876-1878	2017	63	0	0
Innsbruck, Austria	7	10	1881-1883	2010	28	2	0
Prague, Czech Republic	9	10	1954-1967	2014	11	1	0
St. Galmier, France	10	9	1878-1893	2017	22	1	0

Supplementary Table 9. Associations between haplotype frequency and latitude for modern and historic time periods. Emtrends reported trends, standard errors, upper and lower confidence limits alongside contrast statistics of significant two-way interactions identified in Supplementary Data 5, where time is included as a categorical predictor (modern-historic).

Haploblock	Type	Slope estimate	SE	UCL	LCL	Contrast estimate	Contrast SE	Contrast *z*	Contrast *p*
hb-chr2	Historic	0.024	0.019	-0.013	0.061	-0.059	0.026	-2.273	0.023
	Modern	0.082	0.023	0.037	0.128				
hb-chr6b	Europe	0.059	0.023	0.014	0.104	0.074	0.035	2.110	0.035
	North America	-0.014	0.026	-0.065	0.036				
hb-chr16b	Historic	0.082	0.019	0.044	0.119	-0.073	0.026	-2.755	0.006
	Modern	0.154	0.024	0.107	0.202				

Supplementary Table 10. Estimates of hb-chr2 frequency at European latitudinal midpoint.

	Historic	Modern
N	210	156
**2*N***	420	312
**Median year**	1902	2014
**Estimated frequency**	0.37	0.69
**95% CI**	0.30-0.45	0.60-0.76

Supplementary Table 11. Haploblock genotypes with Bonferroni-significant *p*-values from mixed model association with phenotypes.

Haploblock	Phenotype	*β*	*p*-value
hb-chr2	Maximum height	-121.91	7.59E-04
hb-chr2	Stem width	-57.11	8.19E-04
hb-chr5b	Flowering end	10.41	2.22E-03
hb-chr7b	Maximum height	-105.12	1.13E-03
hb-chr16b	50% annual growth day	-5.09	3.97E-04
hb-chr16b	Below ground biomass	-0.58	1.12E-04
hb-chr16b	Female flowering onset	-13.48	1.78E-03
hb-chr16b	Flowering end	-7.26	1.88E-03
hb-chr16b	Flowering onset	-12.33	2.20E-03
hb-chr16b	Maximum height	-142.98	3.81E-05
hb-chr16b	Pollen production end	-8.39	2.16E-05
hb-chr16b	Raceme dying onset	-7.70	8.66E-04
hb-chr16b	Ripe seed onset	-9.30	9.48E-05
hb-chr16b	Shoot biomass	-1697.10	7.46E-04
hb-chr16b	Stem width	-56.15	6.97E-04
hb-chr16b	Total biomass	-2.22	4.13E-04

Supplementary Methods 1. Estimating the strength of selection on hb-chr2 using temporal changes

The putative inversion hb-chr2 increases in frequency between historical and contemporary European populations, consistent with selection favouring its increase in the invasive range. To infer strengths of selection that are sufficient to explain the pattern of frequency change, we considered two simple, deterministic models of selection for the inversion:

- A positive selection model in which the inversion is favoured over the standard haplotype and is eventually expected to fix in European populations
- A balancing selection model in which the increased frequency of the inversion brings it closer to a hypothetical polymorphic equilibrium within the European range.

We note that, while genetic drift will inevitably play some role in the allele frequency dynamics of loci subject to selection (because populations are finite in size), evolutionary dynamics are well-approximated by deterministic models provided the allele frequencies of favourable variants are at least moderately common in the population and selection is strong relative to the inverse of the effective population size[1]. Both assumptions are easily met, and should apply to the hb-chr2 haplotype.

Positive selection model

Let p_t represent the frequency of the hb-chr2 inversion haplotype at an arbitrary generation t. Under a model of positive selection with no dominance, the general solution for the ratio of inversion to standard haplotype frequencies (defined as $x_t = p_t/(1 - p_t)$) is:

$$x_t = x_0(1+s)^t \qquad (\mathbf{1})$$

(*e.g.* [2] pp. 200-203), where s is the fitness increase associated with each copy of the inversion (*i.e.*, the fitnesses of inversion heterozygotes and homozygotes, relative to individuals without the inversion, are 1 + s and $(1 + s)^2$, respectively), and $x_0 = p_0/(1 - p_0)$ denotes the starting frequency at generation $t = 0$. Under this parameterization, the difference in relative fitness between inversion and standard haplotype homozygotes is $2s(1 + s/2) \sim 2s$, with the $2s$ approximation applying when s is small (as we infer below).

The strength of selection is a function of the inversion frequency shift from p_0 to p_t following t generations of evolution:

$$s = \exp\left[\frac{1}{t}\, log\left(\frac{p_t(1-p_0)}{p_0(1-p_t)}\right)\right] - 1 \qquad (\mathbf{2})$$

Since common ragweed is an annual plant, t refers to the number of years that have transpired, and p_0 and p_t can be estimated (with uncertainty) from the contemporary and historical samples.

We used Supplementary Eq. (**2**) to infer strengths of selection (s) that would be consistent with the estimated change in inversion frequencies over time, focusing on the estimated frequencies at the midpoint of the European range (Supplementary Table 10). Inversion frequencies were estimated as p = 0.37 (95% CI = {0.30, 0.45}) in the historic samples, with 1902 representing the median date of samples included in the analysis. For modern samples (sampling date of 2014), the estimated frequency was p = 0.69 (95% CI = {0.60, 0.76}). Given the large sizes of historic and modern collections, and the fact that haploblock frequencies remain intermediate across time, the distance between the 95% CI bounds will be roughly 3.92 standard errors of the frequency estimate. To take into account uncertainty in the frequency estimates, we simulated values of p_t (modern) and p_0 (historic) and used these values, along with Supplementary Eq. (**2**), to generate a distribution of selection coefficients (s) consistent with our data. Specifically, for each time point (i.e., historic or modern), we generated 10^6 allele frequency values by sampling independently from a normal distribution with mean corresponding to the point estimate of the haplotype frequency for the time point (i.e., 0.37 for historic; 0.69 for modern) and a standard deviation of $d/3.92$, where d is the difference between the 95% CI of the estimate for the time point. Our analysis yielded 2 x 10^6 allele frequency values, from which values of s were calculated using Supplementary Eq. (**2**). The estimate and 95% confidence interval for s was calculated directly from the distribution of s values.

Balancing selection model

Although temporal changes in the hb-chr2 haplotype are consistent with changes predicted under the positive selection model presented above, we wished to also evaluate an alternative model in which balancing selection favours evolution of the inversion towards an equilibrium polymorphic state. To explore the strength of selection towards a hypothetical equilibrium within the European range, we considered a simple model of overdominant selection. Note that the overdominant selection model is dynamically equivalent to many other balancing selection models provided the differences in fitness among genotypes are small (consistent with our analysis below). Our results based on the overdominance

model should, therefore, apply more broadly to other scenarios of balancing selection, including scenarios involving negative frequency-dependence and antagonistic pleiotropy[3,4].

Following standard theory (*e.g.*, [5] pp. 270-272), the expected change in frequency over a generation (generation t to generation $t + 1$) is:

$$\Delta p_t = p_{t+1} - p_t = \frac{(s_1 + s_2)p_t(1 - p_t)(p_{eq} - p_t)}{1 - p_{eq}(1 - p_{eq})(s_1 + s_2) - (p_t - p_{eq})^2(s_1 + s_2)} \qquad \mathbf{(3)}$$

where s_1 and s_2 refer to the fitness costs of being homozygous for inversion and standard haplotypes, respectively, and p_{eq} is the equilibrium frequency of the inversion. Using a continuous-time approximation, we can solve for the overall selection coefficient, $S = s_1 + s_2$, that is consistent with a frequency shift from p_0 to $p_t < p_{eq}$ across t generations:

$$t = \int_{p_0}^{p_t} \frac{1 - Sp_{eq}(1 - p_{eq}) - S(x - p_{eq})^2}{Sx(1 - x)(p_{eq} - x)} dx \qquad \mathbf{(4)}$$

$$= \frac{\left(p_{eq} - Sp_{eq}(1 - p_{eq})\right)\log\left(\frac{1 - p_t}{1 - p_0}\right) + (1 - p_{eq})(1 - Sp_{eq})\log\left(\frac{p_t}{p_0}\right) - \left(1 - Sp_{eq}(1 - p_{eq})\right)\log\left(\frac{p_t - p_{eq}}{p_0 - p_{eq}}\right)}{Sp_{eq}(1 - p_{eq})}$$

Solving for S, gives us:

$$S = \frac{p_{eq}\log\left(\frac{1 - p_t}{1 - p_0}\right) + (1 - p_{eq})\log\left(\frac{p_t}{p_0}\right) - \log\left(\frac{p_{eq} - p_t}{p_{eq} - p_0}\right)}{p_{eq}(1 - p_{eq})\left(t + \log\left(\frac{1 - p_t}{1 - p_0}\right) + \log\left(\frac{p_t}{p_0}\right) - \log\left(\frac{p_{eq} - p_t}{p_{eq} - p_0}\right)\right)} \qquad \mathbf{(5)}$$

To infer the strength of selection (S) that would be consistent with observed inversion frequencies and a given equilibrium value (p_{eq}), we simulated 10^6 inversion frequencies consistent with the estimated frequency and its sample size at historical time point (~1902) and 10^6 frequencies consistent with the estimate for the contemporary sample. (Frequencies were simulated as described in the positive selection model section, above). We used each pair of simulated inversion frequencies and Supplementary Eq. (**5**) to infer the value of S consistent with the frequency values. The resulting distribution of 10^6 simulated S values was used to calculate 95% CI for S consistent with the data. We focused on equilibrium values outside of the 95% CI for contemporary inversion frequencies (*i.e.*, values of p_{eq} between 0.80 and 1).

The results show that plausible selection coefficients under scenarios of balancing selection are consistently greater than those of the positive selection model (Supplementary Fig. 15). Selection under the positive selection model can, therefore, be regarded as a lower bound for the strength of selection consistent with the observed temporal changes in European hb-chr2 inversion frequencies.

Supplementary Methods 2. Selection estimated from spatial changes in haploblock frequency

Cline theory

We will consider the simplest possible population genetics model of local adaptation in a species that is continuously distributed along a single axis of space (*e.g.*, from north to south), with x representing location along the axis, and $x = 0$ representing a specific point in space where the environment relevant to selection at a focal locus—in this instance, a genomic region segregating for an inversion—changes abruptly. We assume that the inversion is favoured in locations where $x > 0$ (*e.g.*, in the north) and the standard haplotype is favoured in locations where $x < 0$ (*e.g.*, the south). We further assume that population density is uniform across the spatial gradient (at least within the vicinity of the environmental transition), and that individual dispersal follows a symmetric, Gaussian distribution with variance of σ^2 (the unit of distance is arbitrary, though σ and x should have the same units, *e.g.*: if distance in x is measured in kilometres then σ should also be expressed in km; σ^2 corresponds to the migration rate, m, between adjacent patches in discrete stepping stone models)[6].

Given the stated assumptions, the inversion frequency dynamics at location x can be described using the following reaction diffusion equation:

$$\frac{dp(x)}{dt} = \frac{\sigma^2}{2}\frac{d^2p(x)}{dx^2} + \Delta p_{sel}(x) \qquad \textbf{(6)}$$

where $\Delta p_{sel}(x)$ is the local response to selection[7,8]. With symmetrical strengths of selection at each side of the environmental transition, and no dominance, then $\Delta p_{sel}(x) \approx sp(x)\big(1 - p(x)\big)$ within the northern region of the range where the inversion is favoured, and $\Delta p_{sel}(x) \approx -sp(x)\big(1 - p(x)\big)$ in the southern portion of the range where the standard haplotype is favoured; both expressions are valid for modest-to-weak selection ($0 < s <$ ~0.1). As in the positive selection model presented above, this parameterization leads to local fitness differences of ~2s between inversion and standard haplotypes. Incorporating dominance does not change our results provided the dominance relations between the alleles are consistent across the range (*i.e.*, under "parallel dominance"[9]). At equilibrium between selection and migration, the maximum cline slope will be:

$$\frac{dp(x)}{dx} = \sqrt{\frac{s}{3\sigma^2}} \qquad \textbf{(7)}$$

Following Roughgarden[8], the equilibrium general solution for the cline is:

$$p(x) = -\frac{1}{2} + \frac{3}{2}\left[\tanh\left(x\sqrt{\frac{s}{2\sigma^2}} + \left(\sqrt{\frac{2}{3}}\right)\right)\right]^2 \quad \text{for } x > 0 \tag{8a}$$

$$p(x) = \frac{3}{2} - \frac{3}{2}\left[\tan\left(-x\sqrt{\frac{s}{2\sigma^2}} + \left(\sqrt{\frac{2}{3}}\right)\right)\right]^2 \quad \text{for } x < 0 \tag{8b}$$

Estimating cline slopes by logistic regression

A logistic regression model for inversion frequency as a function of geographic location (x) is:

$$f(x) = \frac{1}{1 + e^{-(\beta_0 + \beta_1 x)}} \tag{9}$$

The parameters of the model (β_0 and β_1) can be estimated by fitting the data to the log-odds (logit):

$$\log\left(\frac{f(x)}{1 - f(x)}\right) = \beta_0 + \beta_1 x \tag{10}$$

Using the theoretical cline functions (above) to calculate the log-odds, we obtain the following slopes. For shallow clines—those with a geographically broad clinal region, where the maximum slope can be accurately estimated—we have:

$$\frac{d\log\left(\frac{p(x)}{1 - p(x)}\right)}{dx} = 4\sqrt{\frac{s}{3\sigma^2}} \tag{11}$$

For steep clines—those with a narrow clinal region, where the maximum slope will be underestimated using the logit function—we have:

$$\frac{d\log\left(\frac{p(x)}{1-p(x)}\right)}{dx} = \sqrt{\frac{2s}{\sigma^2}} \tag{12}$$

We get the following estimates from these two limits:

$$\frac{\sqrt{3}\beta_1}{4} \le \frac{\sqrt{s}}{\sigma} \le \frac{\beta_1}{\sqrt{2}} \tag{13}$$

Given the point estimates and 95% CI for β_1 in Supplementary Data 7, we can calculate plausible ranges for the lower bound of $\frac{\sqrt{s}}{\sigma}$ by multiplying the values for β_1 by $\frac{\sqrt{3}}{4}$

Comparisons of spatially varying selection among haploblocks

All of these estimates rely on the assumption that the system is at equilibrium within each range and time point, though that assumption may be more valid for some cases then others. To the extent that it is a reasonable assumption, and if effects of gene flow are consistent across the genome, we can estimate the relative strength of spatially varying selection on different haploblocks (e.g., haploblocks arbitrarily labeled “A” and “B”) as:

$$\frac{s_A}{s_B} = \left(\frac{\beta_{1,A}}{\beta_{1,B}}\right)^2 \tag{14}$$

Supplementary Methods 3. A simple null model of temporal allele frequency changes under drift

To evaluate whether temporal changes in candidate loci exceeded neutral expectations under drift in the absence of selection, we compared the distribution of the following standardized measure of divergence for a large sample of putatively neutral SNPs with the same metric calculated for selection candidates. Let divergence after t generations be defined as:

$$y_t = \frac{p_t - p_0}{\sqrt{tp_0(1 - p_0)}} \qquad (\mathbf{15})$$

where p_0 and p_t represent the initial and final frequencies of an allele at a bi-allelic locus. We shall show below that, provided loci with low minor allele frequencies are first filtered out of the analysis, the metric follows a symmetric distribution that is approximately independent of the initial frequency.

For a locus with initial frequency of p_0, the frequency after one generation of drift is given by:

$$p_1 = \frac{x}{2N_e} \qquad (\mathbf{16})$$

where x is a random variable drawn from a binomial distribution with parameters $2N_e$ and p_0, where N_e is the effective population size (which follows the standard, Wright-Fisher model of genetic drift). The expected value and the variance for p_1 is therefore p_0 and $p_0(1 - p_0)/2N_e$, respectively. The model can be extrapolated for a modest number of generations, after which the allele frequency (p_t after t generations) has an expected value of p_0 and variance of $tp_0(1 - p_0)/2N_e$. The latter will eventually break down as t increases, but it should be appropriate provided $t/2N_e$ is small and the initial frequency is not too close to zero or one, as we assume below. From these expressions, the standardized measure of allele frequency divergence in the population under drift (and no selection) has an expectation of zero and a variance of:

$$\mathrm{var}(y_t) = \frac{\mathrm{var}(p_t)}{tp_0(1 - p_0)} = \frac{1}{2N_e} \qquad (\mathbf{17})$$

which is independent of the initial frequency.

In reality, error in the estimates of p_0 and p_t will also affect the test statistic, and this will tend to inflate the variance, but doesn't alter the conclusion that (under a null model of drift with no selection) the distribution of the estimates of y_t (which we denote as $\hat{y}_t$) will be roughly independent of the initial allele frequencies in the historic sample. If we define $\hat{p}_t$ and $\hat{p}_0$ is the estimates of the allele frequencies, then our test statistic is:

$$\hat{y}_t = \frac{\hat{p}_t - \hat{p}_0}{\sqrt{t\hat{p}_0(1-\hat{p}_0)}} \qquad (\mathbf{18})$$

The mean and variance of $\hat{y}_t$ can be calculated using the following steps:

Step 1. The expected value and variance of $\hat{p}_t$ conditioned on the final population frequency (*i.e.*, the true frequency, p_t) is:

$$E[\hat{p}_t|p_t] \ = p_t \qquad (\mathbf{19})$$

$$\text{var}[\hat{p}_t|p_t] \ = \frac{p_t(1-p_t)}{n_t} \qquad (\mathbf{20})$$

where n_t represents the number of genes sampled in the contemporary population (*e.g.*, for hb-chr2, 156 individuals were sampled for the contemporary estimate in Europe; given diploidy, we have n_t = 312).

Step 2. The expected value and variance of $\hat{p}_t$ conditioned on the initial population frequency (*i.e.*, the true frequency, p_0) is:

$$E[\hat{p}_t|p_0] \ = E([\hat{p}_t|p_t] \ |p_0) \ = E(p_t|p_0) \ = p_0 \qquad (\mathbf{21})$$

$$\text{var}[\hat{p}_t|p_0] \ = E(\text{var}[\hat{p}_t|p_t] \ |p_0) \ + \text{var}([\hat{p}_t|p_t] \ |p_0) \ = E\left(\frac{p_t(1-p_t)}{n_t}|p_0\right) + \text{var}(p_t|p_0) \qquad (\mathbf{22})$$

$$\text{var}[\hat{p}_t|p_0] \ = \frac{E(p_t|p_0) \ (1-E(p_t|p_0) \ ) - \text{var}(p_t|p_0)}{n_t} + \text{var}(p_t|p_0) \qquad (\mathbf{23})$$

$$= \frac{p_0(1-p_0)}{n_t} + \frac{tp_0(1-p_0)}{2N_e}\left(1-\frac{1}{n_t}\right)$$

where N_e is the effective population size, and n_0 is the number of genes sampled in the historic population.

Step 3. Among loci with an initial frequency estimate of $\hat{p}_0$, the true initial frequency (p_0) will, roughly, follow a distribution with mean and variance of $\hat{p}_0$ and $\hat{p}_0(1-\hat{p}_0)n_0^{-1}$, respectively. Consequently, the expected value and the variance of $\hat{p}_t$ conditioned on the initial frequency estimate $\hat{p}_0$ will be:

$$E[\hat{p}_t|\hat{p}_0] \ = E\{[\hat{p}_t|p_0] \ |\hat{p}_0\} \ = E\{p_0|\hat{p}_0\} \ = \hat{p}_0 \tag{24}$$

$$\begin{aligned} \mathrm{var}[\hat{p}_t|\hat{p}_0] \ &= E\{\mathrm{var}[\hat{p}_t|p_0] \ |\hat{p}_0\} \ + \mathrm{var}\{E[\hat{p}_t|p_0] \ |\hat{p}_0\} \\ &= E\left\{\frac{p_0(1-p_0)}{n_t} + \frac{p_0(1-p_0)}{2N_e}t\left(1-\frac{1}{n_t}\right)|\hat{p}_0\right\} + \mathrm{var}\{p_0 \ |\hat{p}_0\} \end{aligned} \tag{25}$$

$$\mathrm{var}[\hat{p}_t|\hat{p}_0] \ = \ \hat{p}_0(1-\hat{p}_0)\left[\frac{1}{n_t} + \frac{1}{2N_e}t\left(1-\frac{1}{n_t}\right)\right]\left(1-\frac{1}{n_0}\right) + \frac{\hat{p}_0(1-\hat{p}_0)}{n_0} \tag{26}$$

Therefore, the expected value and the variance for $\hat{y}_t$, given an initial frequency estimate of $\hat{p}_0$, will be:

$$E[\hat{y}_t|\hat{p}_0] \ = E\left[\frac{\hat{p}_t - \hat{p}_0}{\sqrt{t\hat{p}_0(1-\hat{p}_0)}}|\hat{p}_0\right] \ = 0 \tag{27}$$

$$\mathrm{var}[\hat{y}_t|\hat{p}_0] \ = \frac{\mathrm{var}[\hat{p}_t|\hat{p}_0]}{t\hat{p}_0(1-\hat{p}_0)} = \frac{\left[\frac{1}{n_t} + \frac{1}{2N_e}t\left(1-\frac{1}{n_t}\right)\right]\left(1-\frac{1}{n_0}\right) + \frac{1}{n_0}}{t} \tag{28}$$

Note that the final expressions are, once again, independent of the initial frequency, though (once again) the pathway to these results requires that $\hat{p}_0$ is not too close to zero or one. Because of this independence, we can pool loci with different initial frequency estimates (with pooling after loci with low minor allele frequency are first removed) to approximate the null distribution for $\hat{y}_t$ as well as the variance of the test statistic: $\mathrm{var}[\hat{y}_t] \ = \mathrm{var}[\hat{y}_t|\hat{p}_0]$.

Incidentally, the expression for $\mathrm{var}[\hat{y}_t|\hat{p}_0]$ can be rearranged by solving for the effective population size across the t generations, *i.e.*:

$$N_e = \frac{\frac{1}{2}t\left(1-\frac{1}{n_t}\right)\left(1-\frac{1}{n_0}\right)}{t\mathrm{var}[\hat{y}_t]\ -\frac{1}{n_0}-\frac{1}{n_t}\left(1-\frac{1}{n_0}\right)} \quad (\mathbf{29})$$

A rough estimate of N_e can be obtained from a set of independent neutral SNPs by using the above formula with the estimated variance of $\hat{y}_t$ substituted for $\mathrm{var}[\hat{y}_t]$.

Simulations

We carried out simulations to test the theoretical predictions of the neutral model presented above, and found that they work well as long as the initial allele frequency estimates are not too close to zero or one ($0.1 < \hat{p}_0 < 0.9$ performs well and $0.2 < \hat{p}_0 < 0.8$ is excellent). Simulations for a given value of $\hat{p}_0$ were carried out using the following steps. First, we used rejection sampling to simulate a distribution of initial population frequencies (p_0) for a given value of $\hat{p}_0$. For each SNP, we sampled a true population frequency (p_0) from a neutral stationary distribution (*i.e.*, a single draw from a symmetric beta distribution with parameters theta = 0.05, which corresponds to the population-scaled mutation rate for the locus)[10]. We then generated a frequency estimate for the SNP from a single draw from a binomial distribution with parameters p_0 and $n_0 = 182$, where n_0 is the number of genes sampled in the historic population. We retained the first 10^4 simulated SNPs whose estimate after binomial sampling matched the focal value of $\hat{p}_0$. From the retained SNPs, we carried out forward Wright-Fisher simulations under pure drift for t generations to determine the contemporary population frequency (p_t) for each SNP. We then carried out a second round of binomial sampling (with parameters p_t and n_t) for each SNP to generate a final allele frequency estimate. The frequency estimates were used to calculate $\hat{y}_t$ for each simulated SNP. Supplementary Fig. 16 shows simulated distributions of $\hat{y}_t$ for different values initial frequency estimates ($\hat{p}_0$). The distributions are roughly independent of $\hat{p}_0$ and their 95% CI are well-approximated by the 95% confidence interval predicted by a normal distribution with variance corresponding to our analytical expression for $var[\hat{y}_t]$ (see above).

Supplementary references

1. Otto, S. P. & Day, T. *A Biologist's Guide to Mathematical Modeling in Ecology and Evolution*. (Princeton University Press, 2011).
2. Hartl, D. L. & Clark, A. G. *Principles of Population Genetics*. (Sinauer, 2007).
3. Connallon, T. & Clark, A. G. Antagonistic versus nonantagonistic models of balancing selection: characterizing the relative timescales and hitchhiking effects of partial selective sweeps. *Evolution* **67**, 908–917 (2013).
4. Chevin, L., Gompert, Z. & Nosil, P. Frequency dependence and the predictability of evolution in a changing environment. *Evolution Letters* **6**, 21–33 (2021).
5. Crow, J. F. & Kimura, M. *An Introduction to Population Genetics Theory*. vol. 26 (The Blackburn Press, 1971).
6. Felsenstein, J. *Theoretical Evolutionary Genetics*. (2019).
7. Haldane, J. B. S. The theory of a cline. *J. Genet.* **48**, 277–284 (1948).
8. Roughgarden, J. *Theory of population genetics and evolutionary ecology: an introduction*. (sidalc.net, 1979).
9. Lasne, C., Sgrò, C. M. & Connallon, T. The relative contributions of the X chromosome and autosomes to local adaptation. *Genetics* **205**, 1285–1304 (2017).
10. Wright, S. Evolution in Mendelian populations. *Genetics* **16**, 97–159 (1931).
