# Supplementary material for: Large haploblocks underlie rapid adaptation in the invasive weed Ambrosia artemisiifolia
Source: Nat Commun. 2023 Mar 27;14:1717. doi: 10.1038/s41467-023-37303-4 (PMC10042993; doi:10.1038/s41467-023-37303-4)
Supplement: Supplementary file 3 — Description of Additional Supplementary Files [file 41467_2023_37303_MOESM3_ESM.pdf]

Description of Additional Supplementary Files

File Name: battlayetal2023-supdata.xlsx
Description: Excel spreadsheet containing Supplementary Data 1-9

Supplementary Data 1: Length of both genome haplotype sequences repeat-masked, by repeat element class (Haplotype 1 CG level 37.84%; bases masked 741,589,487 [66.51%]; Haplotype 2 CG level 37.60%; bases masked 716,386,243 [67.20%]).

Supplementary Data 2: *Ambrosia Artemisiifolia* resequencing sample data, and the use of samples is each analysis.

Supplementary Data 3: Bonferroni-significant GWAS associations, and annotations for the SNPs falling within predicted genes.

Supplementary Data 4: Enriched gene ontology terms within XtX-EAA outlier windows and haploblock regions.

Supplementary Data 5: Reported *F*-values, degrees of freedom and *p*-values of generalised linear models comparing haploblock haplotype frequency to latitude, range, time and any significant interactions. Time in modelled either as discrete timepoints (historic or modern) or as a continuous variable.

Supplementary Data 6: Least squares (LS) means estimates and SE for significant categorical predictors (no interaction effects with latitude) for models where time is a categorical predictor. For each glm the contrasts and slope estimates were adjusted for multiple tests using a FDR correction.

Supplementary Data 7: Slopes, standard errors, lower and upper confidence limits for three-way interactions for glm of haplotype frequency as a function of time, range and their interaction as well as PC1. Slopes from the logistic regression were them used to estimate the strength of selection scaled to dispersal along the cline (sqrt(s)/sigma) using the lower bound (see Estimating cline slopes by logistic regression) by multiplying the slope by sqrt(3)/4.

Supplementary Data 8: Generalised linear model comparing haploblock haplotype frequency at each location and timepoint to latitude, range, time and any significant interactions among them as well as PC1 to control for population structure. Slope estimates, standard errors and their significance is shown. Time is a continuous variable in the model (measured in collection year).

Supplementary Data 9: Slopes, standard errors, lower and upper confidence limits for haploblocks with significant interactions for glm of haplotype frequency as a function of time (continuous variable), range and their interaction as well as PC1. Due to interactions among continuous variables, slopes are estimated at specific values of the other continuous variable.
