# Supplementary material for: Large haploblocks underlie rapid adaptation in the invasive weed Ambrosia artemisiifolia
Source: Nat Commun. 2023 Mar 27;14:1717. doi: 10.1038/s41467-023-37303-4 (PMC10042993; doi:10.1038/s41467-023-37303-4)
Supplement: Supplementary file 5 — Reporting Summary [file 41467_2023_37303_MOESM5_ESM.pdf]

Corresponding author(s): Kathryn A. Hodgins
Last updated by author(s): Mar 7, 2023

Reporting Summary

Nature Portfolio wishes to improve the reproducibility of the work that we publish. This form provides structure for consistency and transparency in reporting. For further information on Nature Portfolio policies, see our Editorial Policies and the Editorial Policy Checklist.

Statistics

For all statistical analyses, confirm that the following items are present in the figure legend, table legend, main text, or Methods section.

n/a	Confirmed	
☐	☒	The exact sample size (*n*) for each experimental group/condition, given as a discrete number and unit of measurement
☐	☒	A statement on whether measurements were taken from distinct samples or whether the same sample was measured repeatedly
☐	☒	The statistical test(s) used AND whether they are one- or two-sided<br>*Only common tests should be described solely by name; describe more complex techniques in the Methods section.*
☐	☒	A description of all covariates tested
☐	☒	A description of any assumptions or corrections, such as tests of normality and adjustment for multiple comparisons
☐	☒	A full description of the statistical parameters including central tendency (e.g. means) or other basic estimates (e.g. regression coefficient) AND variation (e.g. standard deviation) or associated estimates of uncertainty (e.g. confidence intervals)
☐	☒	For null hypothesis testing, the test statistic (e.g. *F*, *t*, *r*) with confidence intervals, effect sizes, degrees of freedom and *P* value noted<br>*Give P values as exact values whenever suitable.*
☐	☒	For Bayesian analysis, information on the choice of priors and Markov chain Monte Carlo settings
☒	☐	For hierarchical and complex designs, identification of the appropriate level for tests and full reporting of outcomes
☐	☒	Estimates of effect sizes (e.g. Cohen's *d*, Pearson's *r*), indicating how they were calculated

Our web collection on statistics for biologists contains articles on many of the points above.

Software and code

Policy information about availability of computer code

Data collection	The raster (3.4-13) package in R (3.6.0) was used to collect WorldClim bioclimatic data.
Data analysis	Genome assembly refinement was performed with GenomeScope (2.0), Jellyfish (2.3.052), AdapterRemoval (2.3.1), BWA-MEM (0.7.17), Picard (2.19.0) and minimap2 (2.17).<br>Genome and transcriptome completeness were assessed with BUSCO (5.1.3).<br>Resequencing read alignment, variant calling, variant filtering and imputation was performed with the Paleomix pipeline (1.2.13.4), AdapterRemoval (2.3.1), BWA (0.7.17), Picard (2.19.0), GATK (3.7), VcfTools (0.1.15) and Beagle (5.2).<br>Genome annotation and related analyses were performed with the IsoSeq pipeline (3), the MAKER pipeline (3.01.03), SNAP (2013-11-29), AUGUSTUS (3.3.3), RepeatMasker (4.1.1), RepeatModeler (2.0.159), ProtExcluder (v.1.2), SnpEff (4.3t), BLAST+ (2.9.0) and R/topGO (2.38.1).<br>Analyses utilizing genotype likelihoods were performed in ANGSD (0.931).<br>PLINK (1.9) and R (3.6.0) were used to prepare genomic data for downstream analyses.<br>XtX analysis was performed in BayPass (2.2).<br>Genotype-environment associations were performed in R (3.6.0).<br>Genome-wide association studies were performed in EMMAX (beta-7Mar2010).<br>Haploblock identification was performed in Lostruct (0.0.0.9000) and statistical modelling of haploblock frequencies was performed in R (3.6.0) using car (3.0-8) and emmeans (1.7.0) packages.<br>Statistical modelling of herbarium specimen phenotypes was performed in R (3.6.0).<br>QTL mapping analysis was performed in Lep-MAP (3) and R (3.6.0).

For manuscripts utilizing custom algorithms or software that are central to the research but not yet described in published literature, software must be made available to editors and reviewers. We strongly encourage code deposition in a community repository (e.g. GitHub). See the Nature Portfolio guidelines for submitting code & software for further information.

Data

Policy information about availability of data

All manuscripts must include a data availability statement. This statement should provide the following information, where applicable:

- Accession codes, unique identifiers, or web links for publicly available datasets
- A description of any restrictions on data availability
- For clinical datasets or third party data, please ensure that the statement adheres to our policy

Sequences used in reference genome assembly and annotation are available from NCBI under BioProject ID PRJNA819156 [https://www.ncbi.nlm.nih.gov/bioproject/PRJNA819156]. The phased diploid genome assembly is available from NCBI under BioProject IDs PRJNA929657 [https://www.ncbi.nlm.nih.gov/bioproject/PRJNA929657] and PRJNA929658 [https://www.ncbi.nlm.nih.gov/bioproject/PRJNA929658]. The haplotype 1 gene annotation GFF file is available from https://doi.org/10.6084/m9.figshare.19672710.v1. Individual sample resequencing data are available from ENA under BioProject IDs PRJEB48563 [http://www.ebi.ac.uk/ena/browser/view/PRJEB48563], PRJNA339123 [http://www.ebi.ac.uk/ena/browser/view/PRJNA339123] and PRJEB34825 [http://www.ebi.ac.uk/ena/browser/view/PRJEB34825]. Source data for figures are provided with this paper [https://doi.org/10.6084/m9.figshare.22207726.v1]. The following publicly available datasets were used in this work: WorldClim bioclimatic variables [https://www.worldclim.org/data/bioclim.html], FLOR-ID flowering time genes [http://www.phytosystems.ulg.ac.be/florid/databases/gene_list/flowering], UniProtKB plants [https://www.uniprot.org/], TAIR10 representative gene model proteins [https://www.arabidopsis.org/download/index-auto.jsp?dir=%2Fdownload_files%2FProteins%2FTAIR10_protein_lists], BUSCO eukaryota odb10 [https://busco.ezlab.org/frames/euka.htm], Global Biodiversity Information Facility database [gbif.org].

Human research participants

Policy information about studies involving human research participants and Sex and Gender in Research.

Reporting on sex and gender	N/A
Population characteristics	N/A
Recruitment	N/A
Ethics oversight	N/A

Note that full information on the approval of the study protocol must also be provided in the manuscript.

Field-specific reporting

Please select the one below that is the best fit for your research. If you are not sure, read the appropriate sections before making your selection.

☐ Life sciences ☐ Behavioural & social sciences ☒ Ecological, evolutionary & environmental sciences

For a reference copy of the document with all sections, see nature.com/documents/nr-reporting-summary-flat.pdf

Ecological, evolutionary & environmental sciences study design

All studies must disclose on these points even when the disclosure is negative.

Study description	This study centres on population-genomic analyses of genetic variation identified from whole-genome sequences of modern and historic A. artemisiifolia samples from North America and Europe previously described in Bieker et al. (2022). Sequences are aligned to a new genome assembly from a sample also described in Bieker et al. (2022). Measurement of phenotypes used in genome-wide association studies are described in van Boheemen Atwater & Hodgins (2019). Digitized images of herbarium samples were additionally phenotyped for stage of flowering.
Research sample	616 whole-genome sequences of mature Ambrosia artemisiifolia individuals (311 modern and 305 historic) sampled to capture the extent of the species' geographic and climatic range in both North America and Europe (details: Supplementary Data 2). 121 modern samples were previously phenotyped by van Boheemen Atwater & Hodgins (2019). All available (985) digitized images of Ambrosia artemisiifolia herbarium samples in GBIF were also phenotyped for stage of flowering. This study also utilized the following datasets: WorldClim bioclimatic variables; FLOR-ID flowering time genes; UniProtKB plants proteins; TAIR10 representative gene model proteins; BUSCO eukaryota odb10 proteins.
Sampling strategy	Historic herbarium samples were sequenced based on availability and to sample the extent of the species' geographic and climatic North American and European ranges. Modern samples were collected and sequenced with the primary aim of sampling the extent of the species' geographic and climatic North American and European ranges, and secondary aims of maximizing within-population sampling, and maximizing the overlap with locations of historic data, all of this within funding constraints. All digitized images of European herbarium samples were phenotyped.
Data collection	Samples for sequencing were provided by herbaria or collected by Bojan Konstantinovic, Bruno Chauvel, François Bretagnolle, Gerhard Karrer, Kathryn A. Hodgins, Lotte A. van Boheemen and Michael Martin (details: see Bieker et al. 2022 table S5). For each

	sample, >50 seeds were collected in a paper lunch bag marked with individual ID, population ID and date. Phenotyping was overseen by Lotte A. van Boheemen and described in van Boheemen Atwater & Hodgins (2019). Digitized images of herbarium samples were downloaded from the Global Biodiversity Information Facility, and phenology was recorded in a spreadsheet by Sam Craig.
Timing and spatial scale	Historic samples utilized in this study were collected between 1830 and 1973. Field collections for modern samples began in 2008 and were repeated yearly based on sample availability until the primary sampling goal (to capture the extent of the species' geographic and climatic range in both North America and Europe) in 2019 (details: Supplementary Data 2).
Data exclusions	Sequences with low coverage and evidence of contamination were excluded.
Reproducibility	The genome resequencing and variant calling was not replicated due to size, complexity and cost. Bioinformatic analyses were performed using all available data and standardized, automated pipelines. The analysis of digitized images of herbarium records was performed using all available samples by a single researcher (Sam Craig) to avoid bias introduced by multiple data collectors.
Randomization	Resequenced samples were grouped in analyses based on sampling location and time period (i.e. historic or modern). Details of randomization in common garden experiments that produced phenotypic data are described in van Boheemen Atwater & Hodgins (2019).
Blinding	Investigators were not blinded to the identity codes of samples. However acquisition of genomic data was performed using standardized, automated pipelines across all samples. Analyses of genomic data were likewise performed using standardized, automated pipelines. The analysis of digitized images of herbarium records was not blinded because sample information is present on the images.

Did the study involve field work? ☐ Yes ☒ No

Reporting for specific materials, systems and methods

We require information from authors about some types of materials, experimental systems and methods used in many studies. Here, indicate whether each material, system or method listed is relevant to your study. If you are not sure if a list item applies to your research, read the appropriate section before selecting a response.

Materials & experimental systems

n/a	Involved in the study	
☒	☐	Antibodies
☒	☐	Eukaryotic cell lines
☒	☐	Palaeontology and archaeology
☒	☐	Animals and other organisms
☒	☐	Clinical data
☒	☐	Dual use research of concern

Methods

n/a	Involved in the study	
☒	☐	ChIP-seq
☒	☐	Flow cytometry
☒	☐	MRI-based neuroimaging
